# Annotations

**Published:** 1906-05-12

**Authors:** 


### ANNOTATIONS.

### Humanitarian or Crank?

The above query can hardly fail to present itself from time to time to dispassionate observers of public events in this country. When, if ever, does the humanitarian overstep the limits of justifiable kindliness and pass into the category of cranks as a result of a loss of his sense of proportion? It may be admitted that humanitarianism, abstractly considered, is a virtue; we have all been brought up to be kind to animals, and ourselves in turn educate those for whom we are responsible along similar lines of conduct. This course is a perfectly natural sequence of our progressing civilisation. On analysis the attitude seems susceptible of reference to a sympathetic sensibility of temperament directly reflecting our own highly organised nervous systems. It is, of course, entirely wrong to credit the brute creation with sensibilities comparable to our own, but we continue to do so in an ever-increasing degree. Followed to its logical conclusion, humanitarianism comes perilously near to a vice. It teaches that physical suffering is the most intolerable of evils, a creed which, in view of the observed facts of nature, places Providence in a most unflattering position and is unquestionably enervating in tendency. That we as a nation are suffering from this tendency is made sufficiently obvious by the prominence of the anti-vivisectionist party. These gentlemen have presented to Parliament a Bill providing for the total abolition of vivisection. This is frankly to place the value of human life on the same plane as that of the brute creation. There is much truth in the assertion that as we become more civilised we incline to place an exaggerated value on life, both human and other. It may be fairly said that the virility of a race is measured by its contempt of physical distresses, a proposition which, if true, has little comfort for us unless we keep a tight rein on our over-kindly propensities.

### The Open-Air League.

This is indeed an age of co-operative effort and of the institution of more or less elaborate organisations devoted to public instruction. The solitary crusader and the passionate pilgrim have for the most part disappeared in favour of leagues and councils, and the cultivation of the "lonely furrow" claims hardly more than an accasional pathetic figure. The conception of an idea is promptly followed by the birth of a scheme having a committee of sponsors and appealing for public subscriptions, which latter may or may not carry an insurance against accidents or other ills. One of the latest movements of the co-operative and educational order is the institution of what is to be known as "The Open-Air League." The title is certainly a refreshing and breezy one, and can hardly excite either suspicion or opposition. The Executive Committee boasts the names of a number of social celebrities, thus affording a guarantee of respectability of a very high order; and, so that the movements of the League may be directed by the highest scientific wisdom, there exists an Advisory Committee, which includes four Fellows of the Royal College of Physicians and an architectural expert of corresponding standing. The programme may be said to centre round the "open window" as an influence in the prevention and cure of disease. Lectures are to proclaim its virtues to those who will attend, and there are pamphlets for those who prefer to sleep at home. But even here the efforts of the League will not terminate. A penetrating scheme, which is to be called "skilled house-to-house visitation," is to be employed, so that even the most callous may be reached and aroused. Public buildings and authorities, not less than the private home and citizen, will be the subject of missionary effort and zeal, and the budding teachers in our training colleges are to be urged to add to their already numerous ambitions the cult of the open window. In addition to all this, the League will concern itself with the economical erection of open-air sanatoria and with the care of patients who, by means of such institutions, have been restored to health and vigour. Altogether, the programme is an attractive one, and the private citizen may ally himself with its expansive aspirations on the usual modest terms.

### The Ethics of Corporal Punishment.

In a recent article on this subject in the "International Journal of Ethics," Mr. Henry S. Salt puts the argument against corporal punishment of every form and degree on very high ground. It is usual to urge that, as a deterrent from evil-doing, the practice is useless, and that, as a matter of fact, experience proclaims its failure. To this Mr. Salt firmly adheres, but he also contends that even were the verdict in the opposite direction corporal punishment ought still to be condemned on the ground of its immorality. In short, the claim is that corporal punishment is useless, and that even when it appears to be effective it is wrong, and the end is gained at too great a cost. In favour of this view is urged a growing detestation and abhorrence of the practice by all social reformers, and the outrage violence inflicts on the sacred supremacy of the human mind and the dignity of the human body. This argument is not only presented against the flogging of criminals, but also against the use of the birch or cane in schools and the parental chastisement of children. The paper is an interesting one, and is informed by high ideals and by a humanitarian motive worthy of general respect and sympathy. But whether the writer will succeed in establishing his central conclusion in the minds of his readers may perhaps be doubted. The world is prone to practical tests, and, as discipline, though not as savage punishment, some forms of bodily suffering seem to carry satisfactory results in a race "semi-civilised," as Mr. Salt proclaims ours to be. Yet he may without much hesitation claim the flowing tide and the future. The history of the past and the growing sentiment of to-day certainly indicate that, whether by the preaching of abstract ethical doctrines, or by the argument from results, corporal punishment will be surely deposed to a lower and lower position.

